# Musical Emotion Perception in Bimodal Patients: Relative Weighting of Musical Mode and Tempo Cues

**DOI:** 10.3389/fnins.2020.00114

**Published:** 2020-02-26

**Authors:** Kristen L. D’Onofrio, Meredith Caldwell, Charles Limb, Spencer Smith, David M. Kessler, René H. Gifford

**Affiliations:** ^1^Cochlear Implant Research Laboratory, Department of Hearing and Speech Sciences, Vanderbilt University, Nashville, TN, United States; ^2^Vanderbilt University Medical Center, Nashville, TN, United States; ^3^Department of Otolaryngology–Head and Neck Surgery, University of California, San Francisco, San Francisco, CA, United States; ^4^Department of Communication Sciences and Disorders, The University of Texas at Austin, Austin, TX, United States

**Keywords:** cochlear implant, bimodal, music perception, musical emotion, hearing loss, frequency following response, spectral modulation detection, psychophysical tuning curve

## Abstract

Several cues are used to convey musical emotion, the two primary being musical mode and musical tempo. Specifically, major and minor modes tend to be associated with positive and negative valence, respectively, and songs at fast tempi have been associated with more positive valence compared to songs at slow tempi (Balkwill and Thompson, 1999; Webster and Weir, 2005). In Experiment I, we examined the relative weighting of musical tempo and musical mode among adult cochlear implant (CI) users combining electric and contralateral acoustic stimulation, or “bimodal” hearing. Our primary hypothesis was that bimodal listeners would utilize both tempo and mode cues in their musical emotion judgments in a manner similar to normal-hearing listeners. Our secondary hypothesis was that low-frequency (LF) spectral resolution in the non-implanted ear, as quantified via psychophysical tuning curves (PTCs) at 262 and 440 Hz, would be significantly correlated with degree of bimodal benefit for musical emotion perception. In Experiment II, we investigated across-channel spectral resolution using a spectral modulation detection (SMD) task and neural representation of temporal fine structure via the frequency following response (FFR) for a 170-ms /da/ stimulus. Results indicate that CI-alone performance was driven almost exclusively by tempo cues, whereas bimodal listening demonstrated use of both tempo and mode. Additionally, bimodal benefit for musical emotion perception may be correlated with spectral resolution in the non-implanted ear via SMD, as well as neural representation of *F*0 amplitude via FFR – though further study with a larger sample size is warranted. Thus, contralateral acoustic hearing can offer significant benefit for musical emotion perception, and the degree of benefit may be dependent upon spectral resolution of the non-implanted ear.

## Introduction

Cochlear implant (CI) technology has improved significantly over the past 30 years, enabling CI users to achieve high levels of speech understanding in quiet listening environments (e.g., average AzBio sentence recognition ranging from 60 to over 80%, even in the absence of visual cues) (Gifford et al., 2018; Gifford and Dorman, 2018; Sladen et al., 2018); however, processing of more complex inputs remains a significant challenge for most CI users (e.g., Hsiao and Gfeller, 2012).

At present, most modern CI processing use an envelope-based strategy in which a fixed pulse rate is amplitude modulated by the envelope of the signal. During this process, the temporal fine structure of the input is discarded. Additional processing limitations include a restricted overall bandwidth (approximately 100–8500 Hz), electrode place mismatch, and spectral smearing. Spectral smearing is particularly problematic and can result from several factors, including a discrete number of electrodes that serve to replace the function of thousands of hair cells, channel interaction due to the inevitable spread of electrical current in a fluid-filled cavity, variable neural survival/degeneration of nerve fibers, and the lack of stochastic neural firing behavior with electrical stimulation. The lack of spectro-temporal detail provided by most CI processing strategies prevents complex signals from being transmitted with accuracy, especially those requiring precise coding of pitch information, such as musical melodies, lexical tone, and vocal emotion (Chatterjee and Peng, 2008; Hsiao and Gfeller, 2012; Luo et al., 2007; Jiam et al., 2017). Thus, music and emotion perception are often significantly poorer in CI users than in normal-hearing listeners.

Music perception ability in CI users is most commonly quantified in terms of four key structural features of music: rhythm, pitch, melody, and timbre. For adult CI patients, performance on temporal-based music tasks tends to be normal or near normal, suggesting minimal to no deficit in tempo or rhythm discrimination (Gfeller et al., 1997; Hsiao and Gfeller, 2012; Kong et al., 2004; Reynolds and Gifford, 2019). In contrast, for the reasons discussed above, pitch, melody, and timbre perception are significantly poorer (Drennan et al., 2015; Kang et al., 2009). We are greatly limited, however, if the assessment of music perception focuses only on the four perceptual elements discussed here. Data from subjective reports add great value to our understanding of music perception in the CI population, with many adult CI users being disappointed with the way music sounds. In fact, several studies report significantly lower music enjoyment ratings post-implantation compared with ratings prior to deafness (Lassaletta et al., 2007; Mirza et al., 2003). Thus, with current technology, a fulfilling sense of music appreciation remains a goal that has yet to be accomplished for many CI recipients.

An additional factor critical to our understanding in this area is the emotional element of music perception. Two primary cues are used to convey musical emotion: musical mode (the type of scale or subset of musical pitches used in the musical excerpt; e.g., major vs. minor) and musical tempo (the speed of the musical excerpt; e.g., fast vs. slow) (Eerola and Vuoskoski, 2013). Specifically, major and minor modes tend to be associated with positive and negative valence, respectively, and songs at fast tempi [i.e., ♩ = 92–196 beats per minute (bpm), Gosselin et al. (2005); ♩ = 80–255 bpm, Peretz et al. (1998)] have been associated with more positive valence compared to songs at slow tempi [i.e., ♩ = 40–60 bpm, Gosselin et al. (2005); ♩ = 20–100 bpm, Peretz et al. (1998), Balkwill and Thompson (1999), Webster and Weir (2005)].

In Western music, a finite set of 12 pitch classes (A, A#/Bb, B, C, C#/Db, D, D#/Eb, E, F, F#/Gb, G, G#/Ab) is utilized, and from these 12 notes, major or minor scales can be produced. The distinction between a major key, e.g., C major, and its parallel natural minor, C natural minor, is a lowered 3rd and 6th scale degree by a half step, or one semitone. For reference, one semitone is the difference between adjacent keys on a keyboard and is the smallest discrete interval utilized in Western music. Normal-hearing listeners can detect changes significantly smaller than one semitone, but the smallest interval detected by CI users is reportedly between 3 and 7.6 semitones, on average (7.6 semitones, Gfeller et al., 2002; 5.7 semitones, Wang et al., 2011; ∼3 semitones, Drennan et al., 2015; Kang et al., 2009). Thus, for CI users, the difference between C major and C minor may be perceptually subtle or even indistinguishable.

To date, only a handful of studies have examined musical emotion perception in CI users, and even fewer have parsed out the degree to which tempo and mode cues are utilized in the CI population. Hopyan et al. (2011) studied musical emotion recognition in children with CIs and found that these individuals were significantly less accurate in their perception of musical emotion than their normal-hearing peers. A limitation of this study, however, was that tempo and mode cues were not varied independently of one another. Thus, it is unclear how the two cues were weighted by these listeners and whether one cue may have dominated their musical emotion judgments.

In order to determine how much weight listeners give to one cue over the other, researchers have begun varying mode and tempo independently of one another. Caldwell et al. (2015) presented stimuli that consisted of clips that were of positive valence (major mode at a fast tempo), of negative valence (minor mode at a slow tempo), and of ambiguous valence (major mode at a slow tempo; minor mode at a fast tempo). They showed that compared to normal-hearing listeners, CI listeners gave significantly more weight to temporal cues (tempo; fast vs. slow) than pitch cues (mode; major vs. minor) when interpreting musical emotion. Specifically, CI users’ ratings of stimuli with the same tempo were similar, irrespective of mode, while normal-hearing listener ratings’ differed significantly for varying mode. Similarly, Hopyan et al. (2016) altered mode, tempo, or both mode and tempo, and found that CI users relied predominantly on tempo. These findings are consistent with previous literature demonstrating that spectral cues are poorly represented for CI users, whereas temporal cues remain robust.

With the known challenges of CI listening, this raises the question of how listeners who utilize the combination of electric (via CI) and acoustic hearing in the contralateral ear (via hearing aid) may perform on tasks of musical emotion perception. The term “bimodal hearing” is conventionally used to refer to the use of a CI in one ear and a hearing aid in the *contralateral ear.* Indeed, bimodal listeners tend to show better objective and subjective music perception outcomes compared with both unilateral and bilateral CI users (Dorman et al., 2008; El Fata et al., 2009; Gfeller et al., 2008; Sucher and McDermott, 2009). This has largely been attributed to improved access to fundamental frequency (*F*0) and low-frequency (LF) fine structure information in the non-implanted ear (e.g., Smith et al., 2002; Moore, 2003; Li et al., 2013; Dincer et al., 2018). However, much less is known about the acoustic benefits to musical emotion perception, particularly for those with significant hearing loss in the non-CI ear.

Shirvani et al. (2016) compared the musical emotion recognition abilities of children with bimodal configurations and unilateral CIs, showing that bimodal listeners performed significantly better than the unilateral CI group, yet still significantly poorer than normal-hearing listeners. However, similar to the study by Hopyan et al. (2011), they did not vary mode and tempo independently of one another. Giannantonio et al. (2015) examined musical emotion perception in 42 children with CIs, 11 of whom were bimodal listeners. These researchers systematically varied mode, tempo, and both mode and tempo cues, and found that the addition of acoustic hearing in the contralateral ear resulted in greater incorporation of the mode cue – a finding that is indicative of better access to important pitch information via acoustic hearing. Still, further research is warranted in the adult population.

The current study is a replication and extension of the previous work by Caldwell et al. (2015) to include bimodal listeners. The purpose was to examine how musical mode cues (major vs. minor) and musical tempo cues (fast vs. slow) influence the perception of musical emotion among bimodal listeners. CI-alone performance was also assessed, thereby allowing a direct comparison to the CI users’ performance in the study by Caldwell et al. (2015), and also allowing for a measure of within-subject bimodal benefit. The primary hypothesis was that, unlike CI-only users, bimodal listeners would utilize both mode and tempo cues in their musical emotion judgments in a manner more similar to normal-hearing listeners. The secondary hypothesis was that LF spectral resolution in the non-implanted ear would be significantly correlated with degree of bimodal benefit for musical emotion perception. Spectral resolution in the non-implanted ear was initially quantified via psychophysical tuning curves (PTCs) (Experiment I). PTCs are considered a psychophysical analog of neural tuning curves, and measure the level of a narrowband noise masker needed to just mask a pure-tone signal fixed in level and in frequency (Moore, 1978). A smaller sample, including a portion from Experiment I, was also tested via spectral modulation detection (SMD) and neural representation of temporal fine structure via the frequency following response (FFR) for a 170-ms /da/ stimulus (Experiment II). In contrast to the within-frequency nature of PTCs, SMD provides an across-frequency measure of spectral resolution. The FFR is an auditory-evoked potential and thereby serves as an objective measure of the auditory system’s spectral resolving capabilities.

## Experiment I

### Method

#### Participants

**FIGURE 1:**
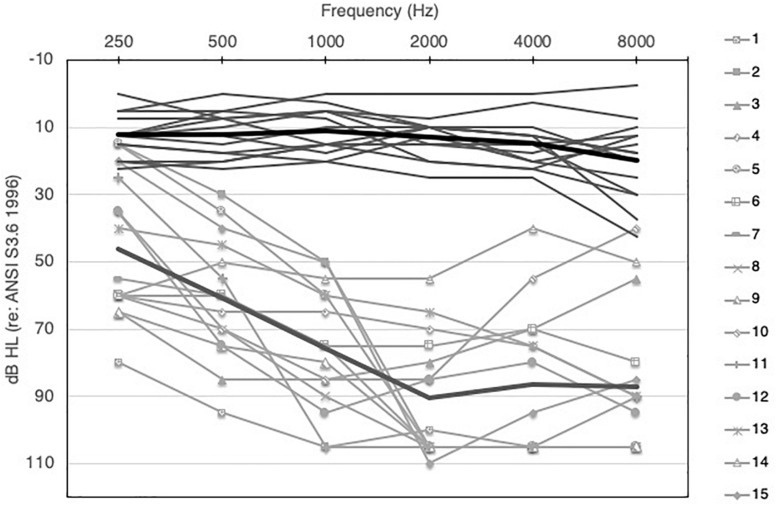
Audiometric thresholds for NH (right and left ears averaged, solid dark gray lines) and bimodal participants (non-implanted ear only, solid light gray lines with symbols). Group means for NH and bimodal are show by the light and dark thick gray lines, respectively.

Participants included 15 adult bimodal listeners and 15 normal-hearing (NH) adult controls. Bimodal listeners ranged in age from 24 to 79 years (mean 56 years), and NH controls ranged in age from 22 to 71 years (mean 47 years). Additional demographic information for the bimodal participants is shown in Table 1. Normal hearing was defined as pure-tone audiometric thresholds ≤25 dB HL from 250 to 4000 Hz, bilaterally. If a hearing evaluation had not been completed within 6 months prior to the study, an audiometric evaluation was performed. A Grason Stadler GSI 61 audiometer with ER-3A insert earphones was used. Audiometric thresholds for both groups are displayed in Figure 1. For the NH group, the right and left ears are averaged together, and for the bimodal group, thresholds are shown for the non-implanted ear only. Although there is significant variability across participants, average hearing loss of the non-implanted ear is moderate sloping to severe. The TEN Test was used to quantify dead regions (areas with few or no functioning inner hair cells and/or auditory neurons) in the non-implanted ear of the bimodal participants (Moore et al., 2000). Dead regions were identified based on a 10-dB or greater shift criterion. Testing using the TEN test determined that 1 participant had a dead region at 500 Hz, 3 at 750 Hz, 1 at 1000 Hz, 3 at 1500 Hz, and 2 at 2000 Hz.

**TABLE 1:** Bimodal participant demographics (Experiment I).

**Participant**	**Age (years)**	**Gender**	**Manufacturer**	**Internal**	**Implant ear**	**Etiology**	**Duration of deafness**	**Strategy**
1	72	M	AB	MidScala	R	Unknown	Longstanding, progressive	Optima-S
2	58	F	Cochlear	CI522	L	Unknown	Unknown	ACE
3	24	F	Cochlear	CI24RE (CA)	R	Unknown	Longstanding, progressive	ACE
4	64	M	AB	MidScala	R	Meniere’s Disease	Longstanding, progressive	Optima-S
5	36	F	AB	MidScala	R	Unknown	Longstanding. progressive	Optima-S
6	35	F	AB	MidScala	L	Unknown	Longstanding	Optima-S
7	79	M	Cochlear	CI24RE (CA)	L	Unknown	Unknown	ACE
8	70	F	AB	MidScala	R	Unknown	Longstanding	Optima-S
9	56	F	AB	MidScala	L	Unknown	Longstanding, progressive	Optima-S
10	54	F	MED-EL	Standard	L	Unknown	Unknown	FS4-p
11	52	M	AB	MidScala	L	Sudden SNHL	Longstanding	Optima-S
12	40	F	Cochlear	CI532	L	Unknown	Unknown	ACE
13	69	M	AB	MidScala	R	Unknown	Longstanding, progressive	Optima-S
14	79	M	Cochlear	CI512	L	Sudden SNHL	5 months	ACE
15	46	F	AB	MidScala	R	Unknown	Unknown	Optima-S
Mean	56							
SD	16.87							

All procedures were explained prior to the study and informed consent was obtained. Following completion of the study, participants were compensated for their participation.

#### Test Environment

All testing was conducted in a single-walled sound-attenuation chamber. All music stimuli were presented at 65 dBA from a single Yamaha Model HS8 powered speaker positioned at 0° azimuth at a distance of 3 meters from the listener. Stimuli were calibrated in terms of sound pressure level at the location of the participant’s head, with the participant absent. For all CI-alone testing in the soundfield, the non-CI ear was plugged with a 3M Classic foam earplug to prevent any inadvertent contribution from the acoustic hearing ear.

#### Cochlear Implant Programming

The CI settings used for testing were those used by the participant in everyday listening. Directional microphone settings were not activated for any of the testing, and CI-aided thresholds were between 20 and 30 dB HL from 250 to 6000 Hz for all participants.

#### Hearing Aid Fitting

Since the primary question of interest focused on bimodal benefit from the hearing aid ear, the authors felt it was important to fit all participants with the same device and hearing aid fitting strategy (e.g., NAL-NL2). This was done in an effort to control factors like compression schemes, signal processing, and other automatic hearing aid features. A Phonak Bolero V90 behind-the-ear (BTE) hearing aid with non-custom comply tip coupling was used for all fittings. All fittings were completed on-ear using Audioscan Verifit’s probe microphone system. The NAL-NL2 hearing aid prescriptive formula was used, and gain targets for 55, 65, and 75 dB SPL input levels were verified. If a match to target within ∼3 dB for all input levels could not be achieved, 65 dB SPL was given priority. Features including noise reduction, acclimatization, frequency lowering, and directional microphone processing were deactivated.

#### Musical Emotion Perception

The musical emotion stimuli in the current study were taken from Caldwell et al. (2015), in an effort to make direct comparisons with their findings. Their stimuli were created with Finale Songwriter 2012 (MakeMusic, Inc. Eden Prairie, MN, United States), and consist of 12 four-bar melodies played on a piano with chordal accompaniment. Each melody included 10 quarter notes, 4 eighth notes, 2 half notes, and a passing chord. All clips were 20 s long. A 2 × 2 design was utilized to generate four variations of the same melody that differed in mode (major vs. minor) and tempo (fast: 180 bpm vs. slow: 60 bpm), thus resulting in either congruent or incongruent pairings of mode and tempo information. The four variations were as follows: Major/Fast (majF) – congruent, Major/Slow (majS) – incongruent, Minor/Fast (minF) – incongruent, and Minor/Slow (minS) – congruent. This resulted in 3 valence categories: positive valence (majF), negative valence (minS), and ambiguous valence (minF and majS). The ambiguous valence stimuli are considered incongruent because the mode and tempo information is conflicting. This category is particularly important because it allows one to examine how participants weight the two cues in their musical emotion perception process. If a difference exists across group/listening configurations (CI-alone vs. bimodal vs. NH) in the degree to which participants weight one cue over the other, it would be evident on the incongruent, ambiguous valence trials. During stimuli presentation, “slow” melodies were played once and “fast” melodies were repeated three times, so that both slow and fast tempo clips were the same overall duration. All four variations of the 12 melodies were presented, yielding 48 test items in total. Task instructions were delivered as follows: “You will hear several short melodies. During each melody, please focus on the emotion conveyed. After the melody is finished, you will be asked to rate it on a scale from 0 (very sad) to 10 (very happy).”

Bimodal listeners were tested in both the CI-alone and bimodal listening configurations. Order of listening configuration was alternated across participants. In order for the NH group to complete the same number of trials as the bimodal group, NH listeners were tested twice. After each 20-s stimulus was played, participants rated the stimulus on a Likert scale from + 5 (very happy) to −5 (very sad).

#### Musical Training and Aptitude Questionnaire

All participants completed the Ollen Musical Sophistication Index (OMSI) (Ollen, 2006) as a measure of individual musical training and aptitude. The OMSI is a 10-item, online questionnaire, which classifies individuals as “more” or “less musically sophisticated.” Specifically, a score is generated which indicates the probability that a music expert would classify that individual as “more musically sophisticated.” Individuals who score over 500 are considered “more musically sophisticated,” and those who score less than 500 are considered “less musically sophisticated” (Ollen, 2006).

#### Psychophysical Tuning Curves

In Experiment I, spectral resolution in the non-implanted ear was quantified in terms of frequency selectivity at 262 Hz (C4, or “middle C”) and 440 Hz (A4, or “A440”), and was measured via PTCs with narrowband noise masker. PTCs were obtained via sweeping psychophysical tuning curve (SWPTC) software (Sęk et al., 2005; Sęk and Moore, 2011). This was completed in each ear individually for participants with normal hearing and in the non-implanted ear of bimodal participants. The bandwidth for the narrowband noise masker was 20% of the signal frequency (Sęk et al., 2005) and all signal and masker parameters were selected as default by the SWPTC program (Sęk and Moore, 2011). The purpose of this measure was to quantify individual LF spectral resolution at two frequencies particularly relevant to the music domain. A440 is considered the tuning standard for music pitch (International Organization for Standardization [ISO], 1975), and both C4 (or “middle C”) and A440, are within the range of the music stimuli utilized in this study. Secondarily, this was completed to examine the relationship between frequency selectivity and bimodal benefit for musical emotion perception.

With the SWPTC software, PTCs are measured using a continuous, narrowband noise masker swept in frequency. Specifically, listeners are asked to detect a pulsed sinusoidal tone in the presence of the masker with a center frequency that sweeps from high to low (reverse sweep) or from low to high (forward sweep). The tone is first presented in the absence of the noise masker to familiarize the listener with the signal of interest. The masker is then added. Instructions to the participant are to press and hold the space bar on a standard computer keyboard when the tone is heard. The masker level is increased at a rate determined by the experimenter (2 dB/sec is the default value) when the space bar is pressed. When the tone is no longer audible, the listener is instructed to release the space bar. The masker level is decreased until the space bar is pressed again, indicating that the tone is again audible. During this process, the level needed to just mask the tone is tracked. In all cases, presentation level for each frequency was determined via the threshold measurement procedure within the software. Once a threshold was determined, the presentation level was calculated to be 10 dB SL.

From this task, a measure of the Q_10_ value and tip frequency of the PTC is estimated. The Q_10_ value indicates sharpness of tuning and is calculated as the signal frequency divided by the bandwidth of the PTC 10 dB above the tip frequency. Higher values indicate sharper tuning and are associated with good frequency selectivity. Lower values indicate broader PTCs and are associated with poorer frequency selectivity, with poorest frequency selectivity approaching a Q_10_ value of 0. From the tip frequency value, tip shift can also be calculated. In this study, tip shift was determined by taking the absolute value of the difference between tip frequency and stimulus frequency. For the NH listeners, right and left ear performance was averaged together for all analyses.

## Data Analysis and Results

Data analysis focused on within-subject rating differences (CI-alone vs. bimodal) and between groups rating differences (bimodal vs. NH, and CI-alone vs. NH). Bimodal benefit was defined as the difference between scores in the bimodal condition and scores in the CI-alone condition. The GraphPad Prism 7.0d (San Diego, CA, United States) and IBM SPSS Statistics Version 25 (Armonk, NY, United States) software programs were utilized for all statistical analyses. For all correlation analyses, the strength of the correlation was quantified using Cohen’s (1988) classification system.

### Musical Emotion Perception

There were two primary analyses of our musical emotion data – the first examined the effect of the mode cue. For this assessment, within-subject comparisons were made across the stimuli pairings for which tempo was held constant (e.g., majF vs. minF, minS vs. majS). The purpose of this analysis was to determine the extent to which listeners were able to make use of mode in their judgments. Said differently, this analysis provided an examination of the extent to which ratings were dominated by the tempo cue. A Wilcoxon matched-pairs signed rank test was used for analysis with the significance level defined as α = 0.05. For all analyses of musical emotion, non-parametric tests were used due to the ordinal nature of the data.

**FIGURE 2:**
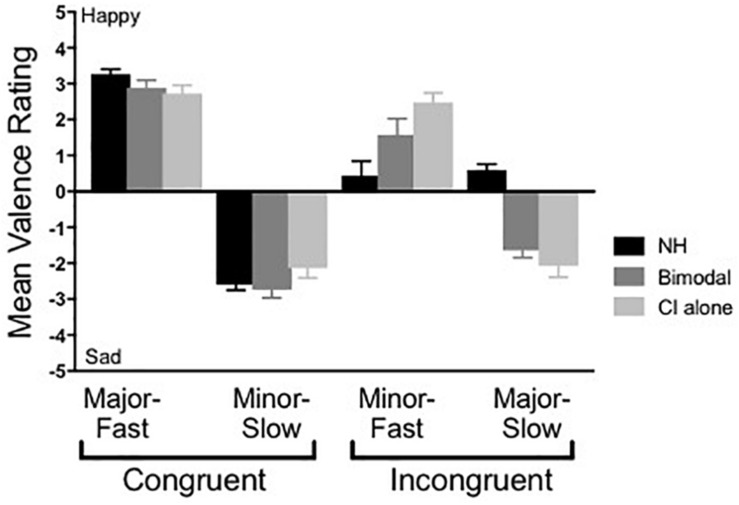
Mean musical emotion ratings across group. Error bars represent + 1 SEM.

Figure 2 shows results from the musical emotion task for the NH listeners and the bimodal participants in both listening conditions. As discussed, the first analysis focused on the degree to which listeners utilized the mode cue. Here, valence ratings were compared across stimuli where the tempo cue was held constant (e.g., majF vs. minF, minS vs. majS). For NH listeners, results from a Wilcoxon matched-pairs signed rank test revealed significant differences in ratings for the majF vs. minF comparison (*Z* = −3.408, *p* = 0.01) and the minS vs. majS comparison (*Z* = −3.408, *p* = 0.01). Likewise, for the bimodal condition, significant differences were evident for both the majF vs. minF comparison (*Z* = −2.926, *p* = 0.01) and the minS vs. majS comparison (*Z* = −3.074, *p* = 0.01). However, for CI-alone, there were no statistically significant differences in ratings for the majF vs. minF comparison (*Z* = −1.609, *p* = 0.11) or the minS vs. majS comparison (*Z* = −0.114, *p* = 0.91). These results demonstrate that when tempo is held constant, NH and bimodal listeners make use of mode in determining the emotional valence of a piece of music, whereas CI-alone listeners do not.

The second analysis focused on the effect of group/listening condition, particularly for the incongruent stimuli pairings. For this assessment, rating differences for both minF and majS were examined, as any benefit of acoustic hearing in the bimodal condition would be evident for these stimuli. Specifically, if bimodal users perceptually combine mode information from acoustic hearing with tempo information through the CI, their emotional valence ratings of incongruent stimuli should be ambiguous, as is observed in NH listeners. A Wilcoxon matched-pairs signed rank test was used to assess differences for CI-alone vs. bimodal, and a Mann-Whitney test was used to assess differences for CI-alone vs. NH and bimodal vs. NH. Significance levels were defined as α = 0.05.

Results from a Wilcoxon matched-pairs signed rank test showed that performance between bimodal and CI-alone listening did not differ significantly for either the minF stimulus (*Z* = −1.917, *p* = 0.06) or the majS stimulus (*Z* = −1.817, *p* = 0.07). Results from a Mann-Whitney test show that the difference in performance between bimodal and NH listeners was not significant for the minF stimulus (*U* = 69, *p* = 0.07), but was significant for the majS stimulus (*U* = 7, *p* = 0.01). The same analysis between CI-alone and NH listeners revealed a significant difference in performance for both the minF stimulus (*U* = 34, *p* = 0.01) and the majS stimulus (*U* = 12, *p* = 0.01). In other words, for the minF stimulus in particular, the addition of acoustic hearing improved CI listener performance to a level that was not significantly different from NH performance. In contrast, performance for the majS stimulus did not demonstrate this same magnitude of improvement for the bimodal listening condition.

### Musical Training and Aptitude Questionnaire

Listeners achieved scores on the OMSI of 441 and 172 for the NH and bimodal groups, respectively. According to the standard OMSI scoring, both groups would be classified as “less musically sophisticated” (Ollen, 2006).

### Psychophysical Tuning Curves

**FIGURE 3:**
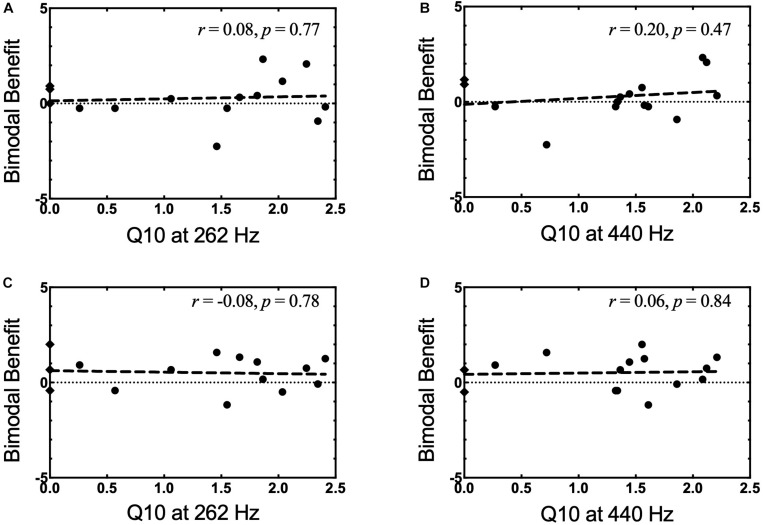
**(A–D)** Bimodal benefit for musical emotion vs. Q_10_. **(A)** minF vs. Q_10_ at 262 Hz. **(B)** minF vs. Q_10_ at 440 Hz. **(C)** majS vs. Q_10_ at 262 Hz. **(D)** majS vs. Q_10_ at 440 Hz. Q_10_ values that could not be completed were represented as a hypothetical Q_10_ value of “0” and notated by a diamond symbol.

Frequency selectivity was evaluated at 262 and 440 Hz, and analysis focused on a comparison of sharpness of tuning, as demonstrated by the Q_10_ value for each frequency. Performance differences for the NH listeners (both ears averaged together) and the non-implanted ear of bimodal patients were compared using an independent sample *t*-test with the significance level defined as α = 0.05. Of note, Q_10_ values for some of the bimodal participants (those with poorest LF thresholds) could not be calculated and were omitted from analysis (Participants 1, 3, and 14 at 262 Hz and Participants 1 and 12 at 440 Hz). Individual Q_10_ values for bimodal participants were included in Figure 3. For the participants for whom a Q_10_ could not be calculated, scores are represented via a hypothetical Q_10_ value of “0” and depicted as a diamond symbol.

Tables 2, 3 show the Q_10_ values and tip shift values for 262 and 440 Hz, respectively, for the NH and bimodal participants. On average, NH listeners demonstrated sharper tuning at both frequencies compared to bimodal listeners. Specifically, the difference in Q_10_ values across groups was significant for both 262 and 440 Hz using an independent sample *t*-test (*t*_25_ = 3.04, *p* = 0.01 and *t*_26_ = 5.65, *p* = 0.01, respectively). This finding was expected given the poorer frequency selectivity and greater variability often seen among listeners with hearing loss (Green et al., 2012). Regarding tip shift, values for NH listeners are expected to be near the test frequency (Sęk and Moore, 2011). Degree of tip shift was minimal and about equivalent for both groups for 262 Hz, and was substantially greater among bimodal listeners for 440 Hz. Our NH results for 440 Hz are consistent with Q_10_ and tip shift values reported for a similar frequency in the literature (e.g., mean Q_10_ at 500 Hz = 2.6, tip shift = 5 Hz, Shabana et al., 2014). Other studies utilizing higher test frequencies have found similar, albeit slightly greater results indicating sharper tuning (e.g., mean Q_10_ at 1000 Hz = ∼4, mean Q_10_ at 4000 Hz = ∼5, Bidelman et al., 2014).

**TABLE 2:** SWPTC results for 262 Hz in NH participants (right and left ears averaged together) and bimodal participants (non-implanted ear only).

	**NH**		**Bimodal**	
	**Q_10_**	**Tip shift (Hz)**	**Q_10_**	**Tip shift (Hz)**
Mean	2.16	10.46	1.61	9.77
SD	0.19	7.09	0.68	11.43

**TABLE 3:** SWPTC results for 440 Hz in NH participants (right and left ears averaged together) and bimodal participants (non-implanted ear only).

	**NH**		**Bimodal**	
	**Q_10_**	**Tip shift (Hz)**	**Q_10_**	**Tip shift (Hz)**
Mean	2.44	7.07	1.50	29.25
SD	0.33	11.72	0.55	31.69

### Relationship Between Bimodal Benefit and Frequency Selectivity

In light of our second hypothesis, the relationship between spectral resolution in the non-implanted ear and bimodal benefit for musical emotion was examined. Pearson product-moment correlations between bimodal benefit for the minF stimulus and Q_10_ at 262 Hz and 440 Hz were both weak and non-significant (*r* = 0.08, *p* = 0.77 and *r* = 0.20, *p* = 0.47, respectively). This relationship is shown in Figures 3A,B, respectively. Pearson product-moment correlations between bimodal benefit for the majS stimulus and Q_10_ at 262 Hz and 440 Hz were also weak and non-significant (*r* = −0.08, *p* = 0.78 and *r* = 0.06, *p* = 0.84, respectively). In other words, there was no statistically significant relationship between spectral resolution – as defined via PTCs – in the non-implanted ear and bimodal benefit for musical emotion perception. This is shown in Figures 3C,D, respectively.

### Relationship Between Bimodal Benefit and Pure Tone Average (PTA)

The relationship between bimodal benefit for musical emotion perception and LF PTA in the non-implanted ear was also examined. LF PTA was defined here as the average of thresholds at 250 and 500 Hz, and ranged from 22.5 dB HL to 87.5 dB HL. Pearson product-moment correlations between LF PTA and bimodal benefit for the minF and majS stimuli were both weak and non-significant (*r* = −0.10, *p* = 0.72 and *r* = −0.14, *p* = 0.63, respectively); thus, audiometric thresholds in the non-implanted ear were not related to bimodal benefit for musical emotion perception.

Because several participants had useable hearing above 500 Hz, both standard PTA (500, 1000, and 2000 Hz) and a high frequency (HF) PTA (4000 and 8000 Hz) were also examined. The Pearson product-moment correlation between bimodal benefit for the minF stimulus and PTA was weak and non-significant (*r* = −0.19, *p* = 0.50), as was the relationship with HF PTA (*r* = −0.07, *p* = 0.82). The Pearson product-moment correlation between bimodal benefit for the majS stimulus and PTA was also weak and non-significant (*r* = −0.30, *p* = 0.27), as was the relationship with HF PTA (*r* = −0.28, *p* = 0.31).

## Discussion

There were two primary questions of interest in Experiment I: (1) Are bimodal listeners able to utilize both mode and tempo cues in their musical emotion judgments in a manner more similar to NH listeners? (2) Is LF spectral resolution in the non-implanted ear, as quantified by PTCs at 262 and 440 Hz, correlated with bimodal benefit for musical emotion perception?

### Are Bimodal Listeners Able to Utilize Both Mode and Tempo Cues in Their Musical Emotion Judgments in a Manner More Similar to NH Listeners?

Our primary question of interest was analyzed in two ways. The first analysis examined the effect of mode, by comparing valence ratings for the stimuli where tempo was held constant (e.g., majF vs. minF, and minS vs. majS). Our data show that both NH and bimodal listeners demonstrated significantly different ratings for the majF vs. minF and minS vs. majS comparisons. In other words, both groups accounted for mode in their ratings of incongruent stimuli. This finding among NH listeners was expected and is consistent with previous literature by Caldwell et al. (2015), where NH listeners provided significantly different ratings for stimuli that varied in mode. Importantly, our findings extend this earlier work to show that with the addition of LF acoustic hearing in the non-implanted ear, bimodal listeners were also able to consider both tempo and mode in their judgments of musical emotion.

In contrast, CI-alone listening relied almost exclusively on tempo cues, as there was no difference in valance ratings for the majF vs. minF and minS vs. majS comparisons. This finding was also consistent with Caldwell et al. (2015), where CI users provided similar ratings to stimuli with the same tempo irrespective of mode. Further, this finding was consistent with existing literature demonstrating that spectral cues are represented poorly among CI users, whereas temporal cues remain robust (Limb and Roy, 2014).

The second analysis focused on the effect of group/listening condition, particularly for the incongruent stimuli pairings. The findings presented here suggest that, on average, bimodal listening yields more typical musical emotion judgments than CI-alone, particularly for the minF stimulus, where ratings in the bimodal condition did not differ significantly from NH ratings. Results for the majS stimulus were also trending similarly, although the improvement was to a lesser degree and remained significantly different from NH performance. This finding is perhaps a product of the participants’ own internal weighting of “slow” vs. “fast.” It is possible that for this group of listeners, a slow tempo is considered more robust with respect to conveying emotion than is a fast tempo, and thus, the slow cue dominated more so than the fast cue for the incongruent pairings. Future research in this area may consider examining and controlling for tempo as an internal weighting factor.

Taken together, these findings yield support for our first hypothesis. The addition of acoustic hearing in the contralateral ear allowed for significantly greater use of the mode cue, and thus, ratings shifted in the direction of NH performance. Further, for the minF stimulus in particular, performance shifted to a degree that was not significantly different from NH performance.

### Is Low-Frequency Spectral Resolution in the Non-implanted Ear Correlated With Bimodal Benefit for Musical Emotion Perception?

Our second question sought to determine whether improvement in the bimodal condition was related to spectral resolution in the non-implanted ear as quantified by frequency selectivity at two frequencies germane to the music domain – 262 Hz and 440 Hz. In contrast to our second hypothesis, mean tuning “sharpness” at 262 and 440 Hz did not appear to be related to bimodal benefit for musical emotion perception, as there was no correlation between bimodal benefit for either minF or majS and frequency selectivity at either frequency.

One interpretation of this finding is that the two frequencies chosen for testing may not have been particularly relevant or generalizable to the specific stimuli utilized in this study. While both frequencies are included in our chordal and melodic stimuli, they account for a relatively small portion of the total notes utilized in these tasks. Alternatively, these frequencies may be partially relevant, but perhaps an analysis of spectral resolution over a broader frequency range would yield better predictive value. SMD was examined in a portion of this sample as a means of quantifying spectral resolution over a broader spectrum and will be discussed as part of Experiment II.

### Relationship Between Bimodal Benefit and PTA

Pure tone average was also not predictive of bimodal benefit for musical emotion perception. This finding carries important clinical significance, as some of the participants with the poorest thresholds received the greatest benefit for musical emotion. It is possible that interpretation of the musical mode cue may rely more heavily upon robust spectral resolution than the mere ability to detect pure tones at a low-level (or even the presence of sharp tuning at two distinct low frequencies). The clinical relevance of this finding is that we likely cannot use the audiogram as a means to determine whether a listener will derive significant musical emotion perception. Clearly, there are contributing factors that have not yet been accounted for.

### Limitations

The authors recognize that there are inherent limitations to using a unidimensional rating scale for the musical emotion judgments. Further, the authors recognize that not all major mode, fast tempo music is perceived as “happy,” and not all minor mode, slow tempo music is perceived as “sad.” There are several additional cues that convey emotion which were not examined in this study, including dynamics, articulation, timing, timbre, consonance/dissonance, and melodic, harmonic, and rhythmic complexity (Balkwill and Thompson, 1999; Gabrielsson and Juslin, 1996). For NH listeners, all of these cues may be utilized for the perception of musical emotion (Bachorowski, 1999; Eerola and Vuoskoski, 2013; Scherer, 2003), whereas for CI users, additional cues involving changes in pitch may be distorted. The stimuli used in this study controlled for most of these additional cues while attempting to isolate the two that are most dominant – mode and tempo. However, in doing so, we may be underestimating CI users’ perception of emotion in more realistic musical pieces. Cues such as dynamics, articulation, timing, and rhythmic complexity can be well-preserved with current signal processing strategies and may be significant contributors to the perception of emotion in real-world music among CI recipients.

## Experiment II

Spectral resolution as measured in Experiment I focused on a within-frequency estimate provided by PTCs. However, as mentioned, spectral resolution at discrete frequencies was likely insufficient for explaining bimodal stimulation relevance for musically complex stimuli as investigated here. Thus in Experiment II, we investigated across-frequency spectral resolution using a SMD task for a broadband carrier (125–8000 Hz). In addition to SMD, we also sought to define the neural representation of periodicity and temporal fine structure via the FFR for a 170-ms /da/ stimulus. The primary question of interest in Experiment II was whether these two measures of spectral resolution may better explain bimodal benefit for musical emotion perception, as compared to the PTC data from Experiment I.

### Method

Participants included 11 NH adult controls and 8 adult bimodal listeners from Experiment I, plus 1 additional bimodal participant who was not included in Experiment I analysis. Bimodal listeners ranged in age from 24 to 79 years (mean 52 years), and NH controls ranged in age from 22 to 71 years (mean 51 years). There were 5 Advanced Bionics users, 3 Cochlear users, and 1 MED-EL user in this sample. Additional demographic information for the bimodal participants is shown in Table 4.

**TABLE 4:** Bimodal participant demographics (Experiment II).

**Participant**	**Age (years)**	**Gender**	**Manufacturer**	**Internal**	**Implant ear**	**Etiology**	**Duration of deafness**	**Strategy**
1	24	F	Cochlear	CI24RE (CA)	R	Unknown	Longstanding, progressive	ACE
2	64	M	AB	MidScala	R	Meniere’s Disease	Longstanding, progressive	Optima-S
3	36	F	AB	MidScala	R	Unknown	Longstanding, progressive	Optima-S
4	79	M	Cochlear	CI24RE (CA)	L	Unknown	Unknown	ACE
5	70	F	AB	MidScala	R	Unknown	Longstanding	Optima-S
6	54	F	MED-EL	Standard	L	Unknown	Unknown	FS4-p
7	40	F	Cochlear	CI532	L	Unknown	Unknown	ACE
8	46	F	AB	MidScala	R	Unknown	Unknown	Optima-S
9*	55	F	AB	MidScala	R	Unknown	Unknown	Optima-S
Mean	52							
SD	17.43							

*Participant was not part of Experiment 1 dataset.

#### Measures of Spectral Resolution

##### Spectral modulation detection

Spectral modulation detection was measured using the quick SMD task developed by Gifford et al. (2014). A three-interval, forced choice paradigm was used based on a modified method of constant stimuli, with two intervals consisting of a flat spectrum noise and the remaining interval consisting of a frequency modulated noise. Unlike the task described previously by Gifford et al. (2014), this version of the task used a constant modulation rate of 1.0 cyc/oct with 10 modulation depths ranging from 4 to 22 dB, in 2-dB steps (Holder et al., 2018). Sixty trials were completed (6 at each modulation depth). A percent correct score for each modulation depth was provided. Stimuli were presented to the non-implanted ear of the bimodal participants at the participant’s most comfortable loudness level (levels ranged from 88–108 dB SPL; mean = 101.79 dB SPL, *SD* = 6). Presentation level across trials was roved ± 5 dB to help avoid level-based cues. NH participants did not complete the SMD task. This measure was added after the study had commenced, and thus, efforts to bring back previously enrolled participants were aimed at bimodal listeners, for whom the relationship between performance and bimodal benefit could be examined.

##### Frequency following response

Frequency following responses were measured using a 170-ms /da/ stimulus (fundamental frequency (*F*0) = 100 Hz, first formant (*F*1) = 700 Hz). Stimuli were delivered at a rate of 4.35 Hz using magnetically shielded Etymotic ER-3A insert earphones in a single-walled sound treated test booth. For the bimodal participants, stimuli were presented at 90 dB SPL to the non-implanted ear. For the NH listeners, stimuli were presented at 80 dB SPL to either the right or left ear (counterbalanced between participants).

Each FFR was taken as the average of 3000 stimulus repetitions, with an artifact rejection of +35 μV. Low-pass and high-pass filters were set to 5000 Hz and 1 Hz, respectively, to permit *post hoc* filtering. Stimulus polarity was set as alternating, and thus allowed for analysis of envelope and temporal fine structure cues by either adding or subtracting responses to each polarity, respectively. An Intelligent Hearing System (IHS) Duet System (Smart EP, Miami FL, United States) was used for stimulus generation and presentation. All participants were positioned in a reclining chair during data collection and were instructed to remain as relaxed as possible, while still remaining awake. A vertical electrode montage with a three Ag-AgCl electrode array (Cz active, Fpz ground, earlobe reference) was utilized. The CI processor was removed during all recordings, and two runs were completed for each participant.

## Data Analysis and Results

### Spectral Modulation Detection

**FIGURE 4:**
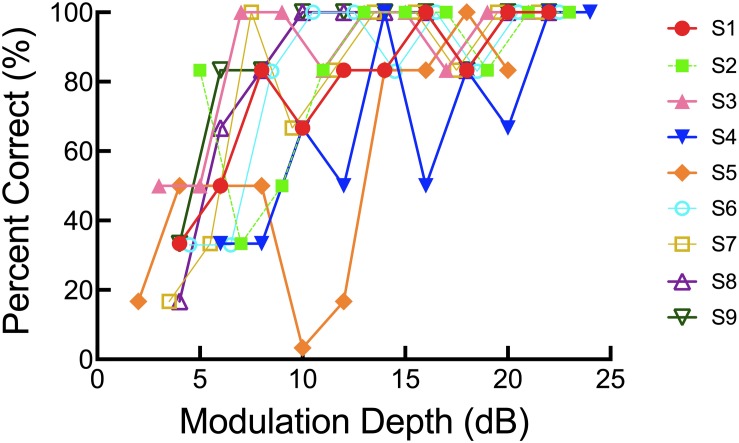
Individual psychometric functions for the SMD task.

Each participant’s percent correct score for each modulation depth was plotted, and a general linear model was used to create a psychometric function. More specifically, the MATLAB statistics toolbox function glmfit was used to generate a logit link function. A threshold (to the nearest dB) was determined for the modulation depth representing the 70% correct point on the psychometric function. Thus, spectral resolution was described as a threshold representing the modulation depth, in dB, yielding 70% correct. Lower thresholds indicate better spectral resolution. On average, acoustic SMD threshold for the non-implanted ear of bimodal listeners was 9.72 dB with a range of 4.56 to 17.43 dB. Individual psychometric functions are plotted in Figure 4.

### Frequency Following Response

The two FFR recordings obtained from each participant were averaged and bandpass filtered between 70 and 3000 Hz. Spectral analysis of the averaged recording was completed using a fast fourier transform (FFT) applied over the 60–180 ms interval of the epoch, which corresponds to the steady state vowel portion of the /da/ stimulus. The envelope of the FFR is unaffected by polarity change, and thus, the FFRs obtained via stimuli of alternating polarities were added in an effort to enhance the *F*0 envelope, while simultaneously reducing the spectral components (e.g., *F*1, *F*2, etc.). The envelope amplitude spectrum at the *F*0 of the /da/ stimulus (100 Hz), in μV, was determined for each participant. *F*0 responses were considered “present” if they were above the estimated noise floor at 100 Hz calculated from the prestimulus interval from −20 to 0 ms. Based on this criterion, energy was present at the *F*0 for all participants in control and bimodal groups.

**FIGURE 5:**
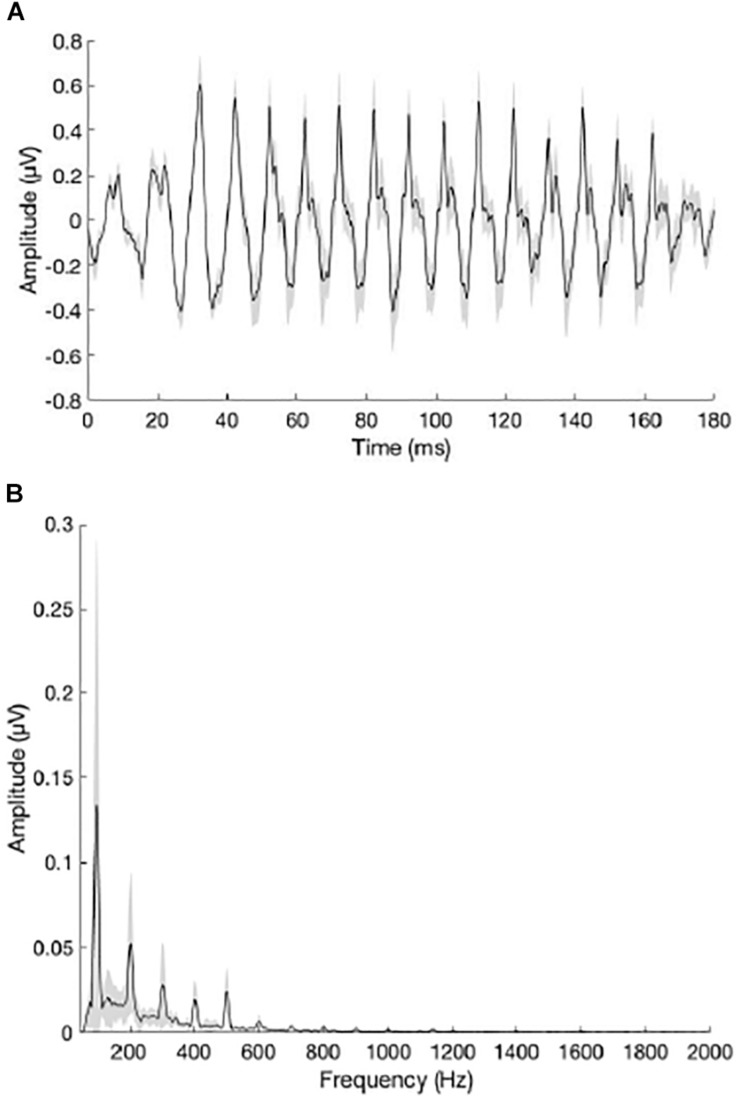
**(A)** Grand average waveform for the NH group. **(B)** Grand average envelope spectrum for the NH group. The peak in the envelope spectrum at 100 Hz reflects neural phase-locking to the *F*0 of the /da/ stimulus. Shading = SEM.

**FIGURE 6:**
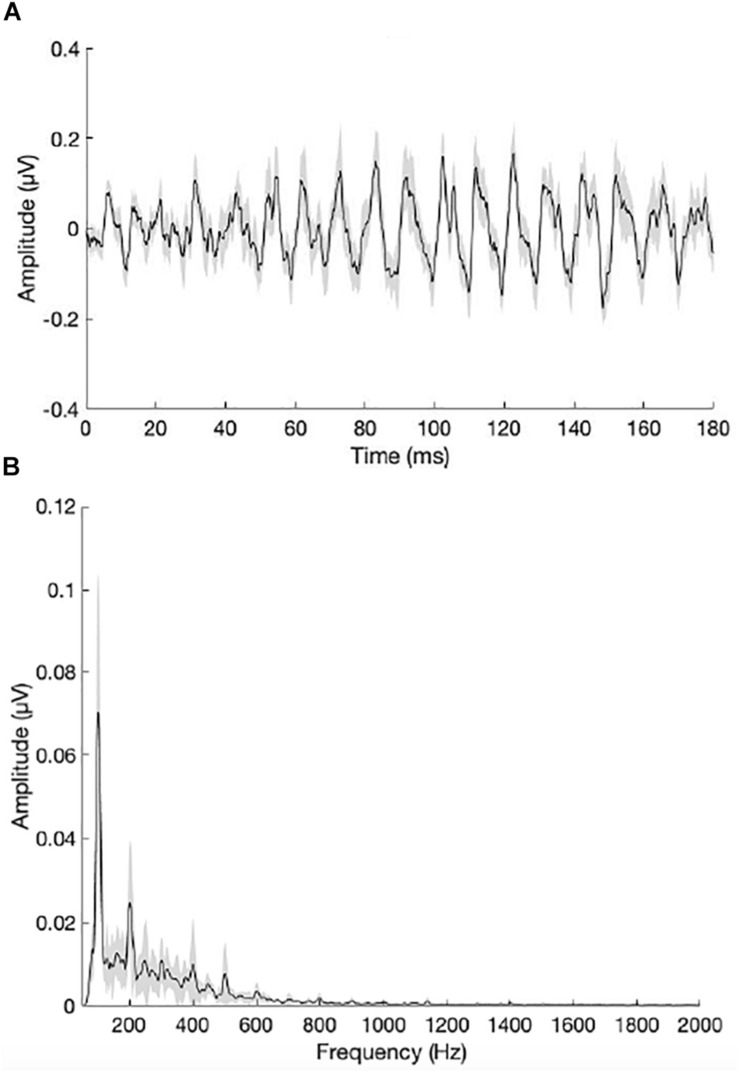
**(A)** Grand average waveform for the bimodal group. **(B)** Grand average envelope spectrum for the bimodal group. The peak in the envelope spectrum at 100 Hz reflects neural phase-locking to the *F*0 of the /da/ stimulus. Shading = SEM.

Figures 5A,B show the grand average waveform and envelope spectra for the NH group. Figures 6A,B show the grand average waveform and envelope spectra for the bimodal group. On average, NH listeners demonstrated larger *F*0 amplitudes compared to bimodal listeners (0.17 μV vs. 0.08 μV, respectively); however, the difference between groups was not statistically significant using an independent sample *t*-test (*t*_11_._12_ = 1.542, *p* = 0.15).

### Relationship Between Bimodal Benefit and Spectral Modulation Detection

**FIGURE 7:**
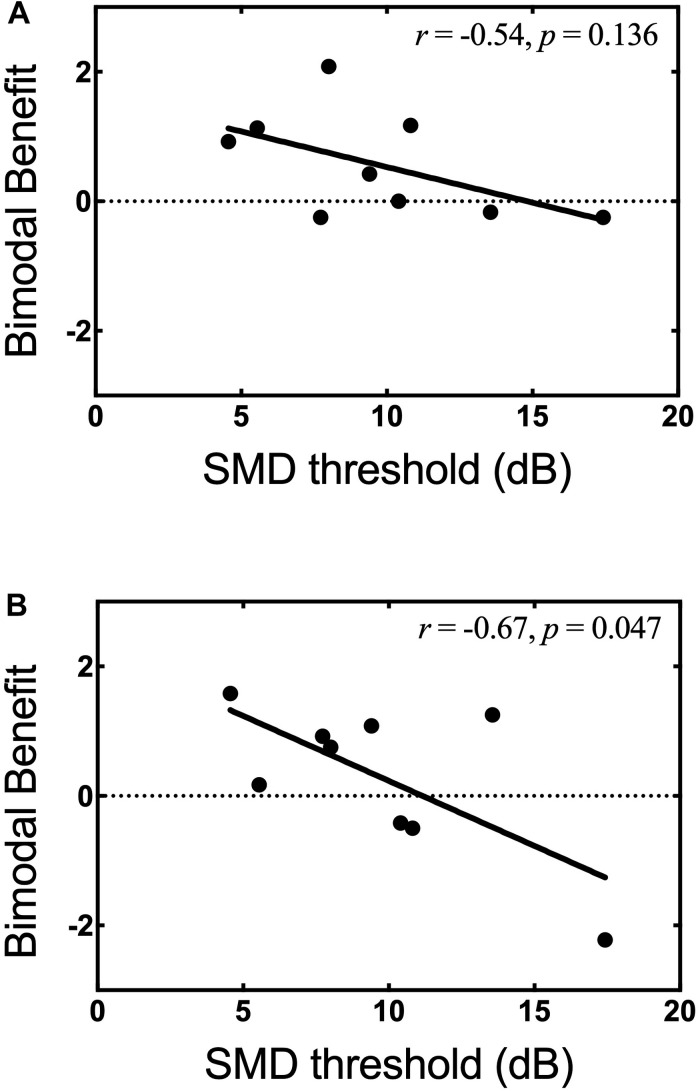
Bimodal benefit for musical emotion vs. SMD threshold. **(A)** minF vs. SMD threshold. **(B)** majS vs. SMD threshold.

In light of our second hypothesis, the relationship between SMD threshold in the non-implanted ear and bimodal benefit for musical emotion was examined. The Pearson product-moment correlation between bimodal benefit for the minF stimulus and SMD threshold was moderate, but not statistically significant (*r* = −0.54, *p* = 0.14). There was a strong correlation between bimodal benefit for the majS stimulus and SMD threshold (*r* = −0.67, *p* = 0.05). These relationships are shown in Figures 7A,B, respectively. Because of the possibility that the majS correlation was driven by a single data point, the relationship with SMD threshold was re-analyzed after removing the participant with bimodal benefit of −2.22 for the majS stimulus. Upon re-analysis, the correlation between bimodal benefit for the majS stimulus vs. SMD threshold was weak and no longer statistically significant (*r* = −0.233, *p* = 0.59).

### Relationship Between Bimodal Benefit and FFR

**FIGURE 8:**
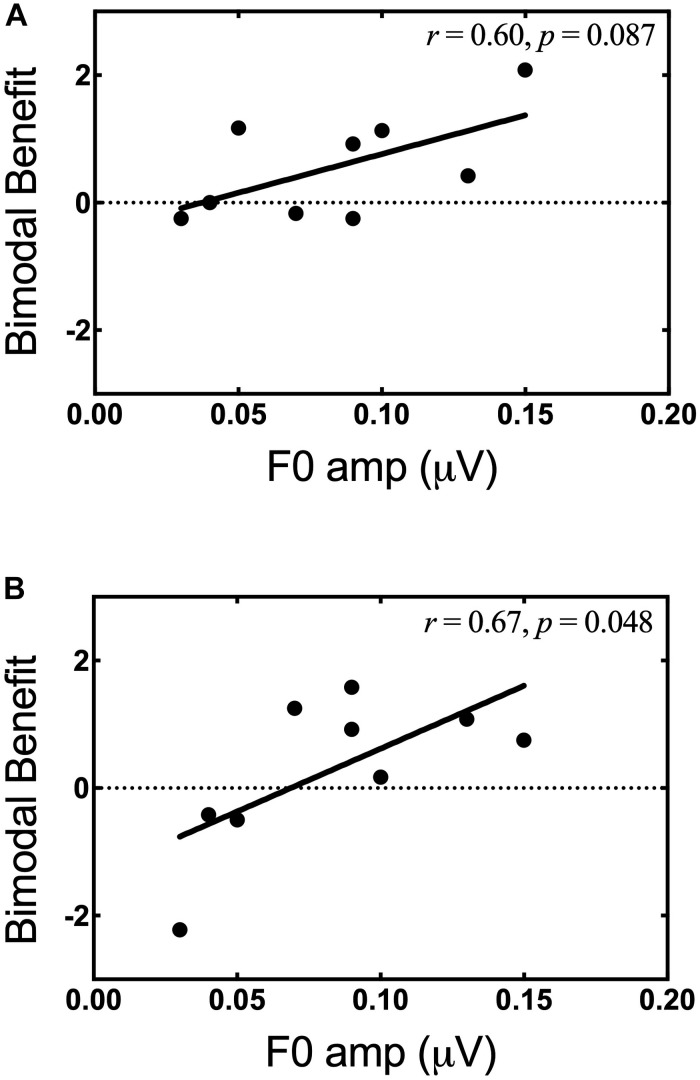
Bimodal benefit for musical emotion vs. *F*0 amplitude. **(A)** minF vs. *F*0 amplitude. **(B)** majS vs. *F*0 amplitude.

The relationship between *F*0 amplitude in the non-implanted ear and bimodal benefit for musical emotion was also examined. Of note, the bimodal listeners did not exhibit evidence for neural representation of *F*1 (700 Hz), likely due to the severity of hearing loss; thus, all analysis was focused on *F*0. The Pearson product-moment correlation between bimodal benefit for the minF stimulus and *F*0 amplitude was moderate, but not statistically significant (*r* = 0.60, *p* = 0.09). In contrast, there was a strong and statistically significant correlation between bimodal benefit for the majS stimulus and *F*0 amplitude (*r* = 0.67, *p* = 0.05). These data suggest that neural representation of *F*0 in the non-implanted ear is related, at least in part, to bimodal benefit for music emotion perception. These relationships are shown in Figures 8A,B, respectively. For the same reasons as discussed in the preceding paragraph, the participant with bimodal benefit of −2.22 for the majS stimulus was removed and the relationship with *F*0 amplitude was re-analyzed. Upon re-analysis, the correlation between bimodal benefit for the majS stimulus vs. *F*0 amplitude was moderate and no longer statistically significant (*r* = 0.524, *p* = 0.18).

## Discussion

For Experiment II, our primary research question was in relation to our secondary hypothesis: Is LF spectral resolution in the non-implanted ear correlated with bimodal benefit for musical emotion perception? Importantly, our results must be interpreted cautiously within the context of the reduced sample size in Experiment II. Future study in this area is warranted. Secondly, Experiment I analyses were rerun with this limited sample, and it is noteworthy that all musical emotion perception results were consistent with the original analyses, with the exception of one comparison. Results from a Wilcoxon matched-pairs signed rank test showed that performance between bimodal and CI-alone listening differed significantly for the minF stimulus (*Z* = −2.193, *p* = 0.03). In other words, this suggests that the addition of acoustic hearing improved performance with minF stimuli to a level that was significantly better than CI-alone. This is the only finding from this smaller sample that differed from Experiment I.

In Experiment II, spectral resolution was quantified behaviorally via SMD and objectively via FFR using a 170-ms /da/ stimulus (*F*0 = 100 Hz). While the FFR is not a conventional measure of spectral resolution, *per se*, it does provide information about the spectral resolving capabilities of the auditory system, as it is a neurophonic response. Our data suggest that both SMD and neural representation of *F*0 amplitude may be correlated with bimodal benefit for musical emotion perception – though further study with a larger sample size is still warranted. Additionally, we should note here that the relationship between SMD thresholds and bimodal benefit for the majS stimulus must be interpreted with caution given that some of the participants exhibited non-monotonic psychometric functions (Figure 5). Still, both SMD and FFR yielded better predictive value than did an examination of within-channel frequency selectivity at 262 and 440 Hz, as discussed in Experiment I.

The results of Experiment II are promising. With respect to the FFR specifically, this measure holds potential for the *objective* assessment of auditory system integrity. Given its objective nature, this is particularly relevant in cases where behavioral responses are unobtainable (e.g., the pediatric population, patients with multiple disabilities, etc.). Furthermore, the FFR may serve as a useful tool in helping to guide clinical recommendation for retention of bimodal hearing or pursuit of a second CI. Indeed, the utility of the FFR as it relates to bimodal benefit extends beyond musical emotion perception, as Kessler et al. (2020) showed a significant relationship between FFR *F*0 amplitude (170-ms /da/) and bimodal benefit for speech recognition. Thus, the FFR appears to hold predictive utility for bimodal benefit in both the speech and music domains. Further investigation is warranted to understand how different features of acoustic speech are neurally encoded in listeners with low frequency residual hearing.

While SMD was also correlated with bimodal benefit for musical emotion perception, Kessler et al. (2020) found no relationship between SMD and bimodal benefit for speech recognition. Therefore, the FFR may be an advantageous measure due to its predictive value with respect to bimodal benefit for both music and speech stimuli. The ability of the FFR to predict speech recognition in noise has been demonstrated across the lifespan in NH listeners and listeners with some hearing loss (Anderson et al., 2010, 2011, 2013; Song et al., 2011; Thompson et al., 2017). Further, an additional advantage is its utility for difficult-to-test populations.

### Limitations

There are important limitations to note for Experiment II. First, as previously discussed, the sample size was small as only a portion of our total sample completed Experiment II. Thus, it is possible that the correlations were driven by a single data point. Indeed, when the participant with bimodal benefit of −2.22 for the majS stimulus was removed from analysis, the correlations between bimodal benefit for the majS stimulus vs. SMD threshold and *F*0 amplitude were weak to moderate and were no longer statistically significant. Further study with a larger sample size is warranted to determine if this data point is truly an outlier, and if the relationships hold for larger groups. Second, hearing loss in the non-CI ear of bimodal participants was variable across participants, and additionally, our approach for determining presentation level for the SMD stimulus was based on the participant’s most comfortable loudness level. Thus, given the variability in hearing sensitivity and the self-selected presentation levels, bandwidth audibility inevitably varied across participants. The individual differences in bandwidth audibility may have differentially affected performance. To better control for variability in audibility, we have interest in future investigations applying frequency-specific amplification for the FFR-stimuli as has been done by Anderson et al. (2013). Finally, while our results show significant promise, an even stronger relationship between bimodal benefit for musical emotion perception and neural representation of periodicity – and also perhaps temporal fine structure – may be shown if a “music” stimulus was used for the FFR recordings (i.e., piano stimulus), as opposed to a “speech” stimulus, as in the /da/ stimulus used here. Future studies may consider examining the same relationship using a musical stimulus for FFR.

## Conclusion

On average, bimodal listeners receive significant benefit from acoustic hearing for musical emotion judgments. Thus, bimodal listening may not only facilitate better music perception, it may also improve musical emotion perception. Two measures of spectral resolution, SMD and FFR *F*0 amplitude for a /da/ stimulus, were significantly correlated with degree of bimodal benefit. Further study is needed with a larger sample size, though both measures may be useful in helping to guide clinical decision-making regarding retention of bimodal hearing or pursuit of a second CI. Conversely, factors such as frequency selectivity at 262 and 440 Hz, musical aptitude and training, and PTA do not appear to be strongly related to bimodal benefit for musical emotion perception. This last point is of significant clinical importance: *benefit does not appear to be related to unaided audiometric thresholds*. Thus, a severe-to-profound hearing loss does not necessarily preclude the possibility of acoustic benefit for music perception and musical emotion perception. Future investigation into the FFR via use of a “music” stimulus should be considered to further examine the relationship with bimodal benefit for musical emotion perception.

## Data Availability Statement

The raw data supporting the conclusions of this article will be made available by the authors, without undue reservation, to any qualified researcher.

## Ethics Statement

This study was carried out in accordance with the recommendations of the Vanderbilt University Institutional Review Board with written informed consent from all subjects. All subjects gave written informed consent in accordance with the Declaration of Helsinki. The protocol was approved by the Vanderbilt University Institutional Review Board.

## Author Contributions

In collaboration with the other co-authors, KD’O was primarily responsible for study development, recruiting participants, collecting and analyzing data, and writing the manuscript. MC and CL developed the musical emotion perception task. SS was a consultant and invaluable resource for all FFR-related methodology, data collection, and data analysis. DK recruited the participants and collected the data. RG is the first author’s Ph.D. mentor, and thus, was an integral part of this project at every stage of this study. MC, CL, SS, DK, and RG all gave input prior to beginning data collection and reviewed and edited the manuscript prior to submission.

## Conflict of Interest

CL is a consultant and research support for Oticon Medical, Advanced Bionics, Med-El Corporation, and is the Chief Medical Officer for Spiral Therapeutics. RG is a member of the eSolutions Advisory Board for Advanced Bionics, Audiology Advisory Board for Cochlear Americas, and the Clinical Advisory Board for Frequency Therapeutics. The remaining authors declare that the research was conducted in the absence of any commercial or financial relationships that could be construed as a potential conflict of interest.
